# Beyond apoptosis: Exploring necroptosis, ferroptosis, and pyroptosis in sevoflurane- and isoflurane-associated PNDs

**DOI:** 10.1016/j.ibneur.2025.09.012

**Published:** 2025-09-24

**Authors:** Haiyan Sun, Rong Cai, Minjuan Zhao, Yisi Shan, Ke Ma, Min Qian

**Affiliations:** a Department of Anesthesiology, Zhangjiagang TCM Hospital Affiliated to Nanjing University of Chinese Medicine, Zhangjiagang, Jiangsu 215600, China; b Translational Medical Innovation Center, Zhangjiagang TCM Hospital Affiliated to Nanjing University of Chinese Medicine, Zhangjiagang, Jiangsu 215600, China; c Department of Neurology, Zhangjiagang TCM Hospital Affiliated to Nanjing University of Chinese Medicine, Zhangjiagang, Jiangsu 215600, China; d Department of Pain Management, Xinhua Hospital Affiliated to Shanghai Jiao Tong University School of Meicine, Shanghai 200092, China

**Keywords:** Perioperative neurocognitive disorders, Anesthetic neurotoxicity, Programmed cell death, Neuroinflammation

## Abstract

Perioperative neurocognitive disorders (PNDs) are common postoperative complications, particularly in elderly patients undergoing general anesthesia. While sevoflurane and isoflurane are widely used inhalational anesthetics, their association with PNDs requires deeper mechanistic investigation. Although apoptosis was traditionally considered the primary mechanism, recent evidence implicates non-apoptotic programmed cell death (PCD) pathways-necroptosis, ferroptosis, and pyroptosis-in PND pathogenesis. These pathways amplify neuroinflammation and neuronal damage through distinct molecular mechanisms. This review synthesizes current understanding of these PCD pathways in inhalational anesthetic-induced neurotoxicity, evaluating their molecular signatures and interactions. We identify critical knowledge gaps and propose research directions focusing on pathway dominance, crosstalk, and multi-target therapeutic strategies. These insights advance PND pathophysiology understanding and inform novel treatment development.

## Highlights

• Non-apoptotic PCD—necroptosis, ferroptosis, and pyroptosis—are emerging as key contributors to anesthetic-induced neurotoxicity in PNDs.
• Crosstalk and dominance among non-apoptotic PCD pathways shape neuroinflammatory responses and neuronal damage following inhalational anesthetic exposure.
• Targeting their shared molecular nodes offers promising multi-target therapeutic strategies for preventing PNDs.

## 1 Introduction

Perioperative neurocognitive disorders (PNDs) encompass a spectrum of cognitive impairments that manifest following general anesthesia and surgery, with particularly high prevalence in geriatric populations (Ye et al., 2025). These disorders, including postoperative delirium (POD) and postoperative cognitive dysfunction (POCD), present clinically as confusion, anxiety, personality alterations, and memory deficits (Niu et al., 2024). Despite their significant clinical impact, the precise etiology of PNDs remains challenging to elucidate, as the relative contributions of surgical trauma, anesthetic agents, and their synergistic interactions are difficult to distinguish.

Sevoflurane and isoflurane, widely utilized inhalational anesthetics, have been extensively investigated for their potential neurotoxic effects, particularly in elderly patients who demonstrate heightened vulnerability to PNDs (Dong et al., 2024, Wang et al., 2024c). The pathophysiology underlying these disorders is multifaceted, involving complex interactions between neuroinflammatory processes, oxidative stress, and neuronal injury (Jia et al., 2023). Elucidating these mechanisms is essential for developing targeted interventions to mitigate PND risk in susceptible populations.

Programmed cell death (PCD), a genetically regulated process fundamental to tissue homeostasis, has emerged as a critical factor in PND pathogenesis. While apoptosis was historically considered the predominant form of PCD involved in anesthetic-induced neurotoxicity (Creeley et al., 2014, Wang et al., 2023d), this mechanism alone cannot adequately explain the robust neuroinflammatory component observed in PNDs. Apoptosis characteristically involves caspase-dependent cellular dismantling with maintenance of membrane integrity, thereby limiting inflammatory responses. In contrast, PNDs exhibit substantial neuroinflammation mediated by pro-inflammatory cytokine release, suggesting the involvement of additional cell death mechanisms.

Recent advances in cell death research have identified several non-apoptotic forms of PCD-notably pyroptosis, necroptosis, and ferroptosis-that contribute significantly to PND pathogenesis (Lin et al., 2025, Liu et al., 2024b, Wang et al., 2023b). These alternative cell death pathways are distinguished by their unique molecular signatures and their propensity to release damage-associated molecular patterns (DAMPs), which amplify inflammatory cascades and exacerbate neuronal injury. The distinct molecular mechanisms governing these pathways not only enhance our understanding of PND pathophysiology but also present novel therapeutic targets for intervention.

This review synthesizes current evidence regarding the roles of non-apoptotic PCD in anesthetic-induced PNDs, with particular emphasis on sevoflurane- and isoflurane-associated neurotoxicity. Through systematic examination of necroptosis, ferroptosis, and pyroptosis in the context of PNDs, we provide a comprehensive mechanistic framework and identify promising directions for future research and therapeutic development.

## 2 The role of necroptosis in sevoflurane- and isoflurane-associated PNDs

Traditionally, PCD was conceptualized exclusively as apoptosis. However, contemporary research has established that necrosis can occur through regulated molecular pathways, termed "programmed necrosis" or "necroptosis" (Degterev et al., 2005). Unlike apoptosis, necroptosis proceeds through caspase-independent mechanisms and exhibits distinctive morphological features, including early plasma membrane permeabilization, cellular edema, and organelle swelling (Zhu and Wu, 2024). This process is orchestrated by a well-defined molecular machinery comprising receptor-interacting protein kinases 1 and 3 (RIPK1 and RIPK3) and mixed lineage kinase domain-like protein (MLKL), which collectively form the necrosome complex (Liu et al., 2021). Upon activation, this complex mediates plasma membrane disruption, resulting in cellular content extravasation and subsequent inflammatory responses.

### 2.1 Necroptosis and neuroinflammation: a bidirectional pathway in PNDs

The relationship between necroptosis and neuroinflammation constitutes a critical bidirectional pathway in anesthetic-induced neurotoxicity (Pasparakis and Vandenabeele, 2015). When neuronal membranes are compromised during necroptosis, various DAMPs-including high mobility group box 1 (HMGB1), ATP, heat shock proteins, and intracellular components-are released into the extracellular environment (Di Virgilio et al., 2023, Dukay et al., 2019, Yang et al., 2021). These DAMPs function as potent activators of pattern recognition receptors (PRRs) expressed by microglia and astrocytes, triggering glial activation and subsequent production of pro-inflammatory mediators such as tumor necrosis factor-α (TNF-α), interleukin-1β (IL-1β), and interleukin-6 (IL-6) (Castellanos-Molina et al., 2024).

This interaction is reciprocal in nature. While necroptosis initiates neuroinflammation through DAMP release, the resulting inflammatory milieu further potentiates necroptosis. Pro-inflammatory cytokines, particularly TNF-α, directly engage the necroptotic machinery by binding to TNF receptor 1 (TNFR1), initiating necrosome formation when caspase-8 activity is compromised (Yuan et al., 2019). This creates a self-amplifying cycle wherein initial anesthetic-induced necroptosis promotes neuroinflammation, which subsequently enhances necroptotic signaling, perpetuating neural damage and cognitive impairment.

In the context of sevoflurane and isoflurane exposure, this necroptosis-neuroinflammation axis appears particularly detrimental in the aging brain, which is characterized by elevated baseline neuroinflammatory status and diminished capacity for inflammatory resolution (Cianciulli et al., 2022, Pluvinage and Wyss-Coray, 2020). This age-dependent vulnerability may partially explain the heightened susceptibility of elderly patients to anesthetic-induced PNDs and suggests that therapeutic strategies targeting either necroptosis or neuroinflammation could potentially interrupt this pathological cycle.

### 2.2 Sevoflurane-induced necroptosis and cognitive dysfunction

**Fig. 1:**
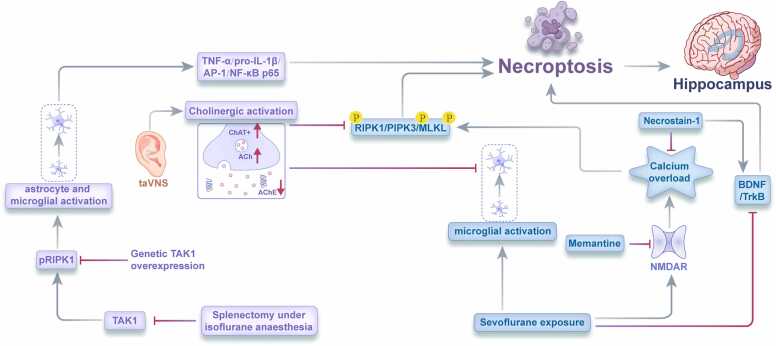
Molecular mechanisms of necroptosis in sevoflurane- and isoflurane-induced PNDs.

Emerging evidence has established necroptosis as a pivotal mechanism in the pathogenesis and progression of sevoflurane-associated PNDs (Fig. 1). Investigations by Wang and colleagues demonstrated that prolonged sevoflurane exposure induces significant cognitive impairment in aged rodents (Yin et al., 2022). Notably, prophylactic administration of necrostatin-1 (Nec-1), a selective RIPK1 inhibitor, significantly attenuated these cognitive deficits. The neuroprotective mechanisms of Nec-1 appear multifaceted, involving inhibition of calcium dysregulation and calpain hyperactivation, modulation of brain-derived neurotrophic factor/tropomyosin receptor kinase B (BDNF/TrkB) signaling cascades, and suppression of necroptotic marker expression in hippocampal neurons.

BDNF, a prominent neurotrophin abundantly expressed throughout the central nervous system, functions as a critical neuroprotective factor essential for neuronal plasticity and cognitive processes including learning and memory (Arévalo and Deogracias, 2023). BDNF exerts its neuroprotective effects predominantly through activation of the TrkB receptor, initiating downstream signaling pathways that promote neuronal survival and synaptic plasticity (Wei et al., 2023). Sevoflurane exposure has been shown to disrupt this BDNF/TrkB signaling axis, contributing to cognitive impairment. The ability of Nec-1 to preserve BDNF/TrkB signaling suggests a mechanistic link between necroptosis inhibition and cognitive protection.

Further research has explored alternative neuroprotective strategies against sevoflurane-induced necroptosis. Transauricular vagus nerve stimulation (taVNS), an innovative neuromodulatory approach approved for epilepsy and depression management (Liu et al., 2024a, Zhang et al., 2023a), has demonstrated efficacy in mitigating sevoflurane-induced cognitive dysfunction in aged rodents. The protective mechanisms of taVNS involve activation of cholinergic anti-inflammatory pathways and reduction of neuronal apoptosis, necroptosis, and microglial activation in the hippocampus (Zhou et al., 2023a). Lesions in the cholinergic basal forebrain abolished these cognitive and neuroprotective effects, highlighting the essential role of cholinergic signaling in taVNS-mediated neuroprotection.

The cholinergic anti-inflammatory pathway represents a crucial neuroimmune mechanism through which taVNS may exert its neuroprotective effects (Ma et al., 2025). Vagal stimulation enhances acetylcholine release, which subsequently binds to α7 nicotinic acetylcholine receptors on immune cells, inhibiting pro-inflammatory cytokine production and attenuating neuroinflammation (Xie et al., 2025). This anti-inflammatory effect, combined with direct inhibition of necroptosis, provides a dual mechanism by which taVNS mitigates sevoflurane-induced cognitive impairment.

Wang's team has recently further elucidated the molecular mechanisms underlying sevoflurane-induced necroptosis (Liu et al., 2024b). The study demonstrated that sevoflurane exposure disrupts calcium homeostasis via N-methyl-D-aspartate (NMDA) receptor hyperactivation, precipitating hippocampal neuron necroptosis. Prophylactic administration of memantine, an NMDA receptor antagonist, significantly reduced cognitive dysfunction in aged mice exposed to sevoflurane. By preventing excessive NMDA receptor activation, memantine inhibits calcium influx, mitochondrial dysfunction, and oxidative stress, thereby preventing necroptosis initiation and preserving neuronal integrity. These findings suggest that targeting upstream mediators of necroptosis may represent an effective strategy for preventing sevoflurane-associated PNDs.

### 2.3 Isoflurane-induced necroptosis and cognitive dysfunction

Isoflurane, another clinically significant inhalational anesthetic, contributes to PND pathogenesis through distinct necroptotic mechanisms (Fig. 1). Zhang and colleagues demonstrated that isoflurane administration during surgical interventions in aged rodents significantly reduced the expression of transforming growth factor β-activated kinase 1 (TAK1), a critical negative regulator of necroptosis (Zhang et al., 2023b). This reduction correlated with increased susceptibility to cognitive dysfunction and neuroinflammation compared to younger subjects, establishing an age-dependent vulnerability pattern.

Mechanistically, TAK1 functions as an endogenous inhibitor of RIPK1, directly phosphorylating RIPK1 to suppress its kinase activity and prevent necrosome formation (Guo et al., 2016). The age-associated decline in TAK1 expression has been causally linked to neurodegenerative conditions-disorders whose symptoms can be ameliorated through RIPK1 inhibition, suggesting a conserved pathophysiological mechanism (Xu et al., 2018). To establish causality, Zhang et al. conducted bidirectional manipulations of the TAK1-RIPK1 axis (Zhang et al., 2023b). Pharmacological inhibition of TAK1 in young rodents significantly exacerbated phosphorylated RIPK1 (pRIPK1) expression, neuroinflammation, and cognitive deficits. Crucially, these deleterious effects were reversed by concurrent administration of RIPK1 inhibitors, confirming RIPK1 as the downstream effector. Conversely, genetic overexpression of TAK1 in aged rodents significantly attenuated pRIPK1 upregulation, neuroinflammation, and cognitive impairment, providing compelling evidence for the TAK1-RIPK1 regulatory axis in isoflurane-associated PNDs.

These findings establish a molecular mechanism whereby age-dependent reduction in TAK1 expression permits aberrant RIPK1 activation following isoflurane exposure, thereby initiating a cascade of neuroinflammation and cognitive dysfunction. This mechanism is particularly relevant in geriatric populations, where baseline TAK1 expression is already compromised. From a translational perspective, the TAK1-RIPK1 regulatory pathway represents a promising therapeutic target for preventing and treating isoflurane-associated PNDs. Pharmacological strategies aimed at either enhancing TAK1 activity or directly inhibiting RIPK1 kinase function may provide effective approaches for mitigating PNDs in vulnerable elderly patients undergoing surgical procedures requiring isoflurane anesthesia.

The identification of necroptosis as a central pathogenic mechanism in both sevoflurane- and isoflurane-associated PNDs has significantly expanded the conceptual framework beyond traditional apoptotic paradigms. The elucidation of specific molecular targets-including RIPK1, RIPK3, MLKL, TAK1, and upstream regulators such as NMDA receptors-provides multiple potential intervention points for developing neuroprotective strategies. While substantial progress has been made in characterizing the molecular mechanisms underlying necroptosis in anesthetic-induced neurotoxicity, several knowledge gaps remain. Further investigation is necessary to fully delineate the temporal dynamics of necroptosis activation, the cell type-specific contributions to PND pathogenesis, and the interactions between necroptosis and other cell death modalities. Addressing these questions will be essential for developing clinically viable interventions to prevent and treat PNDs, ultimately improving perioperative outcomes for surgical patients, particularly those in vulnerable populations.

## 3 The role of ferroptosis in sevoflurane- and isoflurane-associated PNDs

Ferroptosis represents a molecularly distinct form of programmed cell death characterized by iron-dependent accumulation of lipid peroxides (Dixon and Olzmann, 2024). Originally identified by Stockwell and colleagues in 2012, ferroptosis exhibits unique genetic, biochemical, and morphological signatures that differentiate it from apoptosis and other cell death modalities (Dixon et al., 2012). Central to its mechanism is iron-catalyzed lipid peroxidation resulting in oxidative damage to cellular membranes, with reactive oxygen species (ROS) generation as the primary driver (Liu et al., 2025).

### 3.1 Ferroptosis and neuroinflammation: a bidirectional pathway in PNDs

Iron accumulation itself serves as a critical mediator in this ferroptosis-neuroinflammation axis (Li et al., 2024b, Liu et al., 2022). Excessive iron catalyzes the fenton reaction, generating hydroxyl radicals that damage cellular components and activate inflammatory signaling cascades (Lee and Roh, 2025). Iron overload also promotes microglial activation through direct interactions with microglial iron sensors and transporters, shifting their phenotype toward a pro-inflammatory state (Kenkhuis et al., 2023). These activated microglia further contribute to neuronal ferroptosis by releasing pro-oxidant factors and inflammatory mediators, establishing a self-perpetuating cycle of ferroptosis and neuroinflammation (Wang et al., 2023a).

In the context of anesthetic exposure, this ferroptosis-neuroinflammation cycle appears particularly detrimental in the aging brain, which already exhibits dysregulated iron homeostasis, reduced antioxidant capacity, and primed microglia (Gao et al., 2025). Anesthetic exposure further disrupts these already compromised systems, leading to accelerated ferroptosis and exaggerated neuroinflammatory responses. This may partially explain the increased vulnerability of elderly patients to anesthetic-induced PNDs and suggests that therapeutic strategies targeting either ferroptosis or neuroinflammation could potentially interrupt this pathological cycle.

Importantly, neuroinflammation can reciprocally promote ferroptosis through multiple mechanisms (Xu et al., 2024). Pro-inflammatory cytokines downregulate the expression of key ferroptosis defense proteins, including glutathione peroxidase 4 (GPX4) and system xc-, the cystine/glutamate antiporter critical for glutathione synthesis. Activated microglia also release iron-laden ferritin through exocytosis, contributing to the extracellular labile iron pool that can be taken up by neurons, increasing their susceptibility to ferroptosis. Furthermore, inflammatory conditions promote the expression of hepcidin, a key regulator of iron homeostasis that increases cellular iron retention by degrading the iron exporter ferroportin, thereby exacerbating intracellular iron accumulation and ferroptotic vulnerability.

This intricate interplay between ferroptosis and neuroinflammation creates a vicious cycle that amplifies neural damage following anesthetic exposure, particularly in vulnerable populations. Therapeutic strategies that target this cycle at multiple points-by chelating iron, inhibiting lipid peroxidation, enhancing antioxidant defenses, or modulating neuroinflammatory responses-hold significant promise for preventing and treating anesthetic-induced PNDs.

### 3.2 Sevoflurane-induced ferroptosis and cognitive dysfunction

**Fig. 2:**
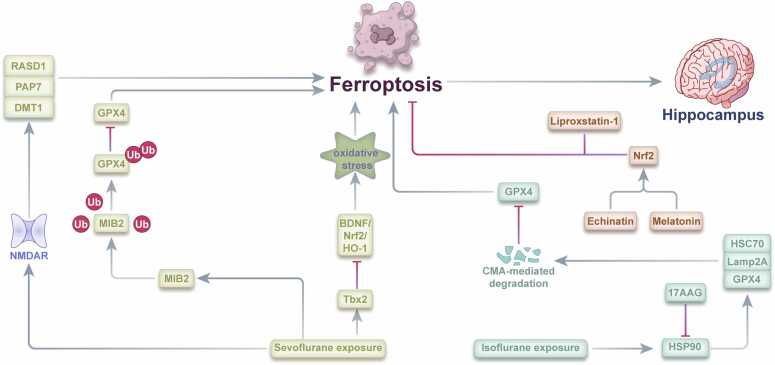
Molecular mechanisms of ferroptosis in sevoflurane- and isoflurane-induced PNDs.

Sevoflurane exposure disrupts iron homeostasis through multiple molecular pathways, ultimately causing neuronal ferroptosis and cognitive impairment (Fig. 2). Wu and colleagues demonstrated that sevoflurane exposure induces iron overload in hippocampal neurons through NMDA receptor/ ras-related dexamethasone-induced 1 (RASD1) signaling, which enhances DMT1-mediated iron uptake (Wu et al., 2020). This iron accumulation initiates ferroptotic cascades, resulting in mitochondrial dysfunction and cognitive deficits that can be mitigated by iron chelation therapy, establishing a causal relationship between iron dysregulation and cognitive impairment.

Molecular investigations have identified several regulatory pathways in sevoflurane-induced ferroptosis. Zhao and colleagues revealed that sevoflurane upregulates Mind bomb-2 (MIB2), an E3 ubiquitin ligase that directly interacts with GPX4, a critical anti-ferroptotic enzyme (Zhao et al., 2021). The MIB2/GPX4 signaling axis modulates hippocampal neuronal vulnerability to ferroptosis following sevoflurane exposure, with MIB2 downregulation significantly reducing ferroptosis.

Further mechanistic insights from Xu and colleagues identified T-Box transcription factor 2 (Tbx2) as a critical mediator of sevoflurane-induced ferroptosis (Xu et al., 2023). Sevoflurane exposure upregulates Tbx2 expression in the hippocampus, concurrent with increased ROS production and lipid peroxidation. Mechanistically, Tbx2 inhibits BDNF expression and subsequent activation of the nuclear factor erythroid 2-related factor 2/heme oxygenase 1 (Nrf2/HO-1) signaling pathway, which normally enhances antioxidant defenses by increasing glutathione synthesis and GPX4 expression.

These mechanistic discoveries have informed targeted therapeutic approaches. Echinatin, a licorice-derived flavonoid, attenuates sevoflurane-induced oxidative stress and ferroptosis through Nrf2 pathway activation (Xu et al., 2022). Similarly, melatonin (Ni et al., 2024) and Liproxstatin-1 (Li et al., 2024a) have demonstrated efficacy in reducing sevoflurane-associated cognitive deficits through antioxidant enhancement and direct ferroptosis inhibition, respectively.

### 3.3 Isoflurane-induced ferroptosis and cognitive dysfunction

Isoflurane similarly induces ferroptosis, albeit through distinct molecular mechanisms (Fig. 2). Zhang and colleagues demonstrated that isoflurane activates the chaperone-mediated autophagy (CMA) pathway in an HSP90-dependent manner, leading to selective degradation of GPX4 and subsequent ferroptosis (Zhang et al., 2024). Heat shock protein 90 (HSP90), typically responsible for protein stabilization during stress (van Oosten-Hawle, 2023), paradoxically facilitates ferroptosis during isoflurane exposure by promoting GPX4 degradation.

Pharmacological inhibition of HSP90 with 17-N-allylamino-17-demethoxygeldanamycin (17-AAG) provides significant neuroprotection against isoflurane-induced neurotoxicity by preserving GPX4 expression and function. This finding establishes HSP90 inhibition as a potential therapeutic strategy for preventing isoflurane-associated PNDs through ferroptosis inhibition. However, complete HSP90 inhibition may induce apoptosis (Okamoto et al., 2008), necessitating the development of selective HSP90 modulators that activate heat shock response without cytotoxicity.

Collectively, these findings establish ferroptosis as a central mechanism in both sevoflurane- and isoflurane-induced neurotoxicity, while identifying multiple therapeutic targets for PND prevention. Strategies aimed at normalizing iron homeostasis, inhibiting ferroptosis directly, and enhancing antioxidant defense systems hold significant promise for mitigating anesthetic-induced cognitive impairment. However, current evidence derives primarily from cellular and animal models, highlighting the need for clinical validation to translate these findings into effective interventions for surgical patients.

## 4 The role of pyroptosis in sevoflurane- and isoflurane-associated PNDs

Pyroptosis represents a molecularly distinct form of programmed cell death characterized by its pro-inflammatory nature. Unlike apoptosis, pyroptosis is characterized by cellular swelling, osmotic lysis, and the release of pro-inflammatory mediators that amplify inflammatory responses (Bai et al., 2025). This inflammatory cell death pathway is initiated primarily through two mechanisms: the canonical pathway involving caspase-1 activation within the inflammasome complex, and the non-canonical pathway mediated by caspase-11 in mice (or caspase-4/5 in humans) in response to intracellular lipopolysaccharides (LPS) (Yu et al., 2021).

### 4.1 Pyroptosis and neuroinflammation: a bidirectional pathway in PNDs

Pyroptosis and neuroinflammation constitute a bidirectional pathophysiological axis that significantly contributes to anesthetic-induced neurotoxicity. This relationship operates through interconnected molecular mechanisms that create a self-perpetuating cycle of neuronal injury and cognitive dysfunction.

The canonical pathway of pyroptosis involves inflammasome activation, particularly the NOD-like receptor protein 3 (NLRP3) inflammasome, which serves as a molecular platform for caspase-1 activation (Xu et al., 2025). Once activated, caspase-1 processes pro-inflammatory cytokines IL-1β and IL-18 into their mature forms and cleaves gasdermin D (GSDMD), generating N-terminal fragments that form membrane pores. These pores facilitate cytokine release and ultimately lead to osmotic cell lysis, releasing additional DAMPs that further activate surrounding immune cells.

This pyroptosis-neuroinflammation relationship is bidirectional in nature (Li et al., 2023). While pyroptosis initiates and amplifies neuroinflammation through cytokine release and DAMP generation, pre-existing neuroinflammatory conditions can reciprocally prime cells for pyroptosis. Pro-inflammatory cytokines such as TNF-α and IL-1β upregulate NLRP3 expression and enhance inflammasome assembly, lowering the threshold for pyroptotic activation. Additionally, inflammatory mediators promote oxidative stress through microglial activation and ROS production, providing the signal for NLRP3 inflammasome activation (Wang et al., 2024b).

The cellular mechanisms linking pyroptosis to neuroinflammation are particularly evident in microglia, the brain's resident immune cells (Yao et al., 2023). Upon anesthetic exposure, microglial NLRP3 inflammasomes are activated, leading to pyroptosis and release of IL-1β and IL-18. These cytokines act on surrounding microglia and astrocytes, propagating inflammatory responses throughout the hippocampus and other cognitive centers. Furthermore, pyroptotic microglial death releases intracellular contents containing various DAMPs, which activate pattern recognition receptors on neighboring cells, creating a spatial spread of inflammation beyond the initial site of cell death (Li et al., 2022).

In the context of anesthetic exposure, this pyroptosis-neuroinflammation cycle demonstrates particular significance in the aging brain, which already exhibits baseline inflammasome priming and microglial activation (Liang et al., 2024). Anesthetic exposure further compromises these already vulnerable systems, accelerating both pyroptosis and neuroinflammation. The resulting amplification of inflammatory signaling disrupts synaptic function and neuronal viability, contributing to cognitive impairment (Wu et al., 2023).

Therapeutic strategies targeting this cycle at multiple points-by inhibiting inflammasome activation, blocking gasdermin pore formation, or modulating upstream regulatory pathways-hold significant promise for preventing and treating anesthetic-induced PNDs.

### 4.2 Sevoflurane-induced pyroptosis and cognitive dysfunction

**Fig. 3:**
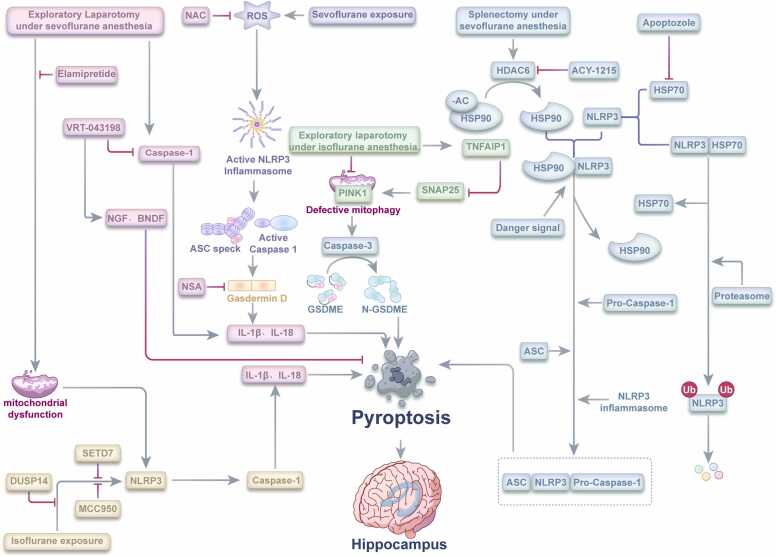
Molecular mechanisms of pyroptosis in sevoflurane- and isoflurane-induced PNDs.

Sevoflurane exposure triggers microglial pyroptosis in the hippocampus through distinct molecular mechanisms, contributing significantly to cognitive dysfunction (Fig. 3). Zhou and colleagues demonstrated that sevoflurane anesthesia induces microglial pyroptosis in the hippocampus of aged mice through ROS-mediated NLRP3 inflammasome activation (Zhou et al., 2023b). Pharmacological inhibition of GSDMD with Necrosulfonamide (NSA) significantly attenuated sevoflurane-induced neurological deficits by preventing pyroptotic pore formation. Similarly, the ROS scavenger N-acetylcysteine (NAC) reduced microglial pyroptosis by suppressing NLRP3 inflammasome activation, confirming the ROS-NLRP3-pyroptosis pathway as a critical mechanism in sevoflurane-associated cognitive decline.

Further mechanistic insights were provided by Lin and colleagues (Lin et al., 2024), who developed a model of POCD using sevoflurane anesthesia followed by splenectomy in aged mice. This study identified histone deacetylase 6 (HDAC6) as a key regulator of microglial pyroptosis through its modulation of heat shock protein 70 (HSP70), HSP90 interactions with NLRP3. HDAC6, primarily known for deacetylating non-histone substrates (Valenzuela-Fernández et al., 2008), facilitates NLRP3 inflammasome assembly and subsequent pyroptosis, contributing to neuroinflammation and cognitive impairment. The therapeutic potential of HDAC6 inhibition has been demonstrated in multiple neurological disorder models (McAlpin et al., 2022, Mondal et al., 2024, Pham et al., 2025), suggesting its relevance as a molecular target for PND prevention and treatment.

Additional research by other investigators demonstrated that caspase-1 inhibition with VRT-043198, a blood-brain barrier-permeable inhibitor, improved cognitive function in aged mice subjected to abdominal exploratory laparotomy under sevoflurane anesthesia (Tang et al., 2022). This improvement was attributed to reduced pyroptosis-associated inflammation and restored expression of neurotrophic factors including nerve growth factor (NGF) and brain-derived neurotrophic factor (BDNF), essential for cognitive function and neuronal survival.

### 4.3 Isoflurane-induced pyroptosis and cognitive dysfunction

Isoflurane anesthesia similarly induces pyroptosis through NLRP3 inflammasome activation, albeit with distinct molecular features (Fig. 3). Fan and colleagues demonstrated that isoflurane exposure activates the NLRP3 inflammasome in neurons, leading to caspase-1 activation, inflammatory cytokine release, and subsequent pyroptosis (Fan et al., 2018). Pretreatment with the NLRP3 inflammasome inhibitor MCC950 significantly reduced NLRP3 expression, cleaved caspase-1 levels, and pro-inflammatory cytokine production, protecting aged mice from isoflurane-induced neuronal damage and cognitive decline. This study was the first to establish pyroptosis as a critical mechanism in isoflurane-induced cognitive impairment and highlight the therapeutic potential of NLRP3 inflammasome inhibition.

Further mechanistic investigations have identified key regulatory molecules in isoflurane-induced pyroptosis. Que and colleagues demonstrated that Dual-specificity phosphatase 14 (DUSP14) inhibits NLRP3 inflammasome-dependent pyroptosis, serving as a crucial regulator in mitigating neuronal injury and cognitive impairment in aged rats (Que et al., 2020). DUSP14, a member of the mitogen-activated protein kinase (MAPK) phosphatase family, functions as a negative regulator of inflammatory responses across various conditions (Wang et al., 2018, Wang et al., 2017, Zhu et al., 2024). These findings propose DUSP14 as an upstream regulator of the NLRP3 inflammasome-caspase-1 pathway, presenting a novel therapeutic target for isoflurane-associated PNDs.

Similarly, Ma and colleagues identified SET domain containing 7 (SETD7), a lysine methyltransferase, as a positive regulator of NLRP3 inflammasome activation and subsequent pyroptosis in isoflurane-exposed aged mice (Ma et al., 2022). SETD7 inhibition effectively reduced neuroinflammation, pyroptosis, and cognitive impairment by suppressing NLRP3 inflammasome activation, further confirming the central role of inflammasome-dependent pyroptosis in isoflurane-induced neurotoxicity.

In the clinical context, where anesthesia and surgery occur simultaneously, Zuo and colleagues demonstrated that isoflurane anesthesia during laparotomy caused mitochondrial dysfunction, triggered the NLRP3 inflammasome-caspase-1 pyroptosis pathway, and reduced synaptic integrity proteins in the hippocampus of aged mice (Ma et al., 2022). The mitochondrial-targeted peptide elamipretide provided protection against these effects and reduced cognitive deficits, underscoring the importance of mitochondrial function in regulating pyroptotic pathways during anesthesia and surgery.

Recent investigations by Wang and colleagues have elucidated alternative pyroptotic mechanisms involving PTEN-induced kinase 1 (PINK1)-mediated mitophagy and the caspase-3/GSDME pathway (Wang et al., 2023c). Their studies revealed that PINK1-dependent mitophagy regulates cognitive impairment induced by laparotomy under isoflurane anesthesia by modulating caspase-3 activation and subsequent GSDME-mediated pyroptosis. Further exploration identified tumor necrosis factor α-induced protein 1 (TNFAIP1) as a ubiquitin ligase that facilitates the degradation of synaptosomal-associated protein 25 (SNAP25), thereby impairing PINK1/Parkin-dependent mitophagy and enhancing caspase-3/GSDME-driven pyroptosis (Wang et al., 2023b).

Collectively, these findings establish pyroptosis as a central mechanism in both sevoflurane- and isoflurane-induced neurotoxicity, while identifying multiple therapeutic targets for PND prevention. Strategies aimed at inhibiting inflammasome activation, blocking gasdermin pore formation, or modulating upstream regulatory pathways hold significant promise for mitigating anesthetic-induced cognitive impairment. However, current evidence derives primarily from preclinical models, highlighting the need for clinical validation to translate these findings into effective interventions for surgical patients.

## 5 Molecular crosstalk between non-apoptotic cell death pathways in PNDs

While necroptosis, ferroptosis, and pyroptosis have been traditionally studied as discrete cell death modalities, emerging evidence reveals significant molecular crosstalk between these pathways in the context of anesthetic-induced PNDs. Understanding these interactions is crucial for developing comprehensive therapeutic strategies that address the complex pathophysiology of PNDs.

### 5.1 Shared signaling nodes and pathway convergence

At the molecular level, these pathways converge on several key signaling nodes that integrate diverse cellular stressors into coordinated cell death responses. Oxidative stress represents a central convergence point, with ROS serving as both initiators and effectors across all three pathways (Liu et al., 2023). In necroptosis, RIPK1/RIPK3-mediated activation of metabolic enzymes generates mitochondrial ROS that amplify cell death signaling (Yuan et al., 2019). Similarly, ferroptosis is fundamentally characterized by iron-dependent lipid peroxidation and subsequent ROS accumulation (Endale et al., 2023). In pyroptosis, ROS production serves as a critical signal for NLRP3 inflammasome activation (Wang et al., 2024a). This shared dependence on redox homeostasis creates bidirectional amplification loops where ROS generated through one pathway can accelerate execution of the others.

Mitochondrial dysfunction constitutes another critical intersection (Bock and Tait, 2020, Chen et al., 2023a). While traditionally associated with apoptosis, mitochondria emerge as central regulators of all three non-apoptotic death pathways. RIPK3-mediated phosphorylation of the mitochondrial phosphatase PGAM5 during necroptosis promotes mitochondrial fragmentation (Cheng et al., 2021). Ferroptosis execution involves mitochondrial membrane lipid peroxidation and subsequent bioenergetic collapse (Fu et al., 2023). In pyroptosis, mitochondrial damage releases mitochondrial DNA and cardiolipin, which serve as DAMPs that activate the NLRP3 inflammasome (Li et al., 2020). This convergence on mitochondrial integrity suggests that mitochondrial-targeted interventions may simultaneously modulate multiple cell death pathways.

### 5.2 Sequential activation and pathway switching mechanisms

Recent investigations reveal that instead of functioning in isolation, the initial activation of one pathway can facilitate subsequent execution through alternative mechanisms (Eskander et al., 2025). For instance, moderate activation of the necroptotic machinery through RIPK1/RIPK3 can induce mitochondrial dysfunction without immediate cell death, subsequently depleting glutathione and increasing cellular susceptibility to ferroptosis. Similarly, inflammasome activation during early pyroptotic signaling increases cellular ROS production, which can deplete antioxidant defenses and sensitize neurons to ferroptotic death. This sequential activation explains why interventions targeting single pathways often provide only partial neuroprotection against anesthetic-induced cognitive deficits.

Molecular switches that determine cell fate decisions between death modalities have been identified. The availability of caspase-8 represents one such switch point (Pang and Vince, 2023). when active, caspase-8 cleaves and inactivates RIPK1/RIPK3, preventing necroptosis and favoring apoptosis. However, under caspase-inhibitory conditions frequently observed during anesthetic exposure, this suppression is relieved, redirecting cell death toward necroptosis. Similarly, the cellular iron status and availability of lipid peroxidation substrates can shift the balance between pyroptosis and ferroptosis in response to anesthetic agents.

### 5.3 Integrated inflammatory signaling networks

The inflammatory consequences of these cell death pathways exhibit substantial overlap and synergistic amplification. While pyroptosis is inherently pro-inflammatory through gasdermin-mediated cytokine release (Wei et al., 2022), both necroptosis and ferroptosis also generate robust inflammatory responses through distinct mechanisms (Chen et al., 2023b, Dhuriya and Sharma, 2018).

This inflammatory crosstalk is particularly evident in microglia-neuron interactions during anesthetic exposure (Suo et al., 2025). Microglial activation through any of these pathways triggers production of pro-inflammatory cytokines that compromise neuronal viability and synaptic function. Conversely, neuronal death through these mechanisms releases DAMPs that further activate microglia, creating a self-sustaining inflammatory cycle that persists beyond the initial anesthetic exposure.

## 6 Conclusion and perspective

PNDs represent a significant clinical challenge with substantial implications for patient outcomes and healthcare systems. This comprehensive review has systematically examined the potential roles of three major non-apoptotic programmed cell death pathways—necroptosis, ferroptosis, and pyroptosis—in sevoflurane- and isoflurane-associated neurotoxicity. Our analysis suggests several critical insights that may advance our understanding of PND pathogenesis and identify potential avenues for therapeutic intervention, though clinical validation remains limited.

### 6.1 Integration of multiple cell death pathways in PND pathogenesis

The evidence presented in this review indicates that anesthetic-associated neurotoxicity involves a complex interplay among multiple cell death modalities rather than proceeding through a single pathway. While apoptosis was historically considered the predominant form of neuronal death following anesthetic exposure, our analysis reveals that non-apoptotic mechanisms may contribute significantly to the neuroinflammatory and neurodegenerative processes underlying PNDs.

Each cell death pathway exhibits distinct molecular signatures yet shares common features that can lead to bidirectional amplification loops. Necroptosis, executed through RIPK1/RIPK3/MLKL signaling, releases DAMPs that activate inflammatory cascades. Ferroptosis, characterized by iron-dependent lipid peroxidation, generates oxidative stress that compromises cellular integrity. Pyroptosis, mediated through inflammasome activation and gasdermin pore formation, directly propagates inflammatory signaling. Together, these pathways may establish a self-sustaining cycle of cellular damage and inflammation that persists beyond the initial anesthetic exposure.

The molecular crosstalk between these pathways occurs at multiple levels—from shared upstream triggers like calcium dysregulation and mitochondrial dysfunction to convergent downstream consequences including membrane disruption and DAMP release. This interconnectedness may explain why interventions targeting single pathways often result in only partial neuroprotection, highlighting the need for therapeutic strategies that simultaneously address multiple cell death mechanisms.

### 6.2 Age-dependent vulnerability and personalized intervention strategies

A consistent finding across studies is the heightened vulnerability of the aging brain to anesthetic-induced non-apoptotic cell death. This age-dependent susceptibility stems from multiple factors: baseline neuroinflammatory status, compromised antioxidant defenses, dysregulated iron homeostasis, and primed inflammasome activation. These pre-existing conditions lower the threshold for initiating cell death cascades following anesthetic exposure, potentially contributing to the increased incidence of PNDs in geriatric populations.

This observation supports the notion that preventive and therapeutic approaches should be tailored to patient-specific risk profiles. Elderly patients may benefit from more aggressive anti-inflammatory and antioxidant interventions, while pediatric patients might require strategies focused on preserving neurodevelopmental processes. The development of clinically accessible biomarkers—such as plasma levels of MLKL oligomers (necroptosis), GPX4 (ferroptosis), or GSDMD-N (pyroptosis)—should be prioritized to enable real-time monitoring of pathway activation and guide risk-stratified interventions in perioperative settings. However, the sensitivity and specificity of these markers require validation in prospective clinical cohorts.

### 6.3 Translational challenges and future research directions

Despite substantial progress in understanding the molecular mechanisms of non-apoptotic cell death in PNDs, significant translational challenges remain. Most evidence derives from cellular and animal models, with limited direct validation in human patients, raising concerns about clinical relevance. To bridge this gap, future studies must prioritize the validation of pathway-specific biomarkers in well-characterized patient cohorts undergoing anesthesia, particularly in aged individuals who are most at risk. Such biomarkers are essential for enabling early detection, real-time monitoring, and personalized intervention in the perioperative period.

Future research should focus on several key areas:

(1) Temporal dynamics of cell death pathways: Well-designed longitudinal studies in rodent models exposed to sevoflurane or isoflurane are critically needed to delineate the temporal sequence of pathway activation (e.g., early pyroptosis triggering ferroptosis via ROS, followed by necroptosis amplification). Elucidating this cascade will define precise therapeutic windows for targeted inhibition—such as administering MCC950 during the early inflammatory phase and ferrostatin-1 during the peak of oxidative stress.
(2) Cell type-specific contributions: Advanced multi-omics approaches, including single-cell RNA sequencing and spatial transcriptomics, should be applied to brain tissues from anesthetic-exposed models to dissect the distinct roles of neurons, microglia, and astrocytes in each death pathway. For instance, given the evidence that microglial pyroptosis drives neuronal ferroptosis via cytokine release, genetically engineered cell-specific knockout models (e.g., Gsdmd-conditional KO in microglia) are warranted to validate causal relationships and identify cell-autonomous versus non-cell-autonomous mechanisms.
(3) Pharmacological modulators: While inhibitors like necrostatin-1 and ferrostatin-1 show efficacy in preclinical models, their poor pharmacokinetic properties and limited blood-brain barrier penetration hinder translation. Therefore, future efforts should focus on developing next-generation analogs with improved CNS bioavailability and target specificity—particularly for MLKL and GPX4—using rational, structure-based drug design approaches.
(4) Combination therapies: Given the robust synergistic amplification between pathways, the co-administration of low-dose, pathway-selective inhibitors (e.g., RIPK1 inhibitor + lipophilic antioxidant) should be systematically evaluated in aged animal models to maximize neuroprotection while minimizing toxicity and off-target effects. This strategy may overcome the limitations of monotherapies in complex, multifactorial conditions like PNDs.
(5) Long-term consequences: Longitudinal cognitive assessments combined with post-mortem analysis of cell death markers in animal models are essential to establish causal links between acute pathway activation and persistent cognitive deficits—a fundamental requirement for validating these mechanisms as clinically relevant therapeutic targets.

Furthermore, while this review focuses on sevoflurane and isoflurane due to their widespread clinical use and robust evidence base in PNDs, future comparative mechanistic studies should extend to other inhalational anesthetics, such as desflurane and nitrous oxide. A systematic, side-by-side evaluation of their differential effects on non-apoptotic cell death pathways—particularly in aged or vulnerable models—is imperative to inform evidence-based anesthetic selection and optimize perioperative neuroprotective management.

### 6.4 Therapeutic implications and clinical applications

The mechanistic insights presented in this review suggest several promising therapeutic approaches for preventing and treating anesthetic-induced PNDs:

(1) Pathway-specific inhibitors: Compounds like necrostatin-1 (necroptosis), ferrostatin-1 (ferroptosis), and MCC950 (pyroptosis) have demonstrated efficacy in preclinical models and warrant further evaluation in clinically relevant models and early-phase human studies.
(2) Mitochondrial-targeted interventions: Given the central role of mitochondrial dysfunction across all three pathways, mitochondrial-targeted antioxidants and metabolic modulators may represent viable therapeutic candidates.
(3) Anti-inflammatory strategies: Interventions that interrupt the inflammatory amplification loops between cell death and neuroinflammation could offer neuroprotection across multiple pathways.
(4) Perioperative management protocols: Evidence-based guidelines for anesthetic selection, dosing, and duration based on patient-specific risk factors could help reduce PND incidence in vulnerable populations.
(5) Combination approaches: Multi-modal interventions targeting complementary aspects of cell death signaling may provide synergistic neuroprotection compared to single-pathway inhibition, though potential interactions and safety profiles require careful assessment.

### 6.5 Concluding remarks

This review highlights the central role of necroptosis, ferroptosis, and pyroptosis—and their interconnectivity—in mediating sevoflurane- and isoflurane-induced neurotoxicity. The convergence of mitochondrial dysfunction, oxidative stress, and neuroinflammation creates a self-amplifying loop that drives neuronal damage, particularly in the aging brain.

Rather than pursuing broad neuroprotective claims, future research should focus on rigorously validating the causal roles of specific molecular nodes—such as MLKL phosphorylation, GPX4 degradation, and GSDMD cleavage—in clinically relevant models. Translation will depend on developing reliable biomarkers and pharmacologically tractable inhibitors with favorable CNS profiles.

By anchoring therapeutic development in the mechanistic framework outlined here, we may move closer to targeted, evidence-based interventions that could help reduce anesthetic-related neurocognitive risk in vulnerable populations. However, the path from mechanistic insight to clinical application remains long and requires sustained collaboration between basic scientists and clinicians.

## Funding statement

This work was supported by the Suzhou Health Youth Core Talent "National Mentorship" Training Project (Qngg2024034), Zhangjiagang Health System Youth Science and Technology Project (ZJGQNKJ202313), Zhangjiagang Medical and Health Science and Technology Innovation Guidance Project (ZKYL2218, ZKYL2441), Suzhou Science and Technology Development Plan Project (SKYD2023056), and the Key project of University-land collaborative Innovation Research Project of Jiangsu Vocational College of Medicine in 2023 (20239608).

## CRediT authorship contribution statement

**Haiyan Sun:** Writing – review & editing, Writing – original draft, Funding acquisition. **Minjuan Zhao:** Writing – review & editing, Writing – original draft. **Rong Cai:** Writing – review & editing, Writing – original draft, Funding acquisition. **Ke Ma:** Supervision, Funding acquisition. **Yisi Shan:** Writing – review & editing. **Min Qian:** Supervision, Funding acquisition.

## Declaration of Competing Interest

The authors declare that they have no known competing financial interests or personal relationships that could have appeared to influence the work reported in this paper.

## Data Availability

No datasets were generated or analysed during the current study.
